# Supplementary material for: A cost-effective predictive tool for AFP-negative focal hepatic lesions of retrospective study: enhancing clinical triage and decision-making
Source: PeerJ. 2025 Mar 26;13:e19150. doi: 10.7717/peerj.19150 (PMC11954459; doi:10.7717/peerj.19150)
Supplement: Supplemental Information 2 — Independent risk factors, including age, gender, ALT levels, HBV/HCV infection status, LMR, and single lesion presence, were identified to construct a predictive model. This model aims to reduce the 3-6 month waiting time for follow-up and facilitate rapid clinical decision-making. [file peerj-13-19150-s002.pdf]

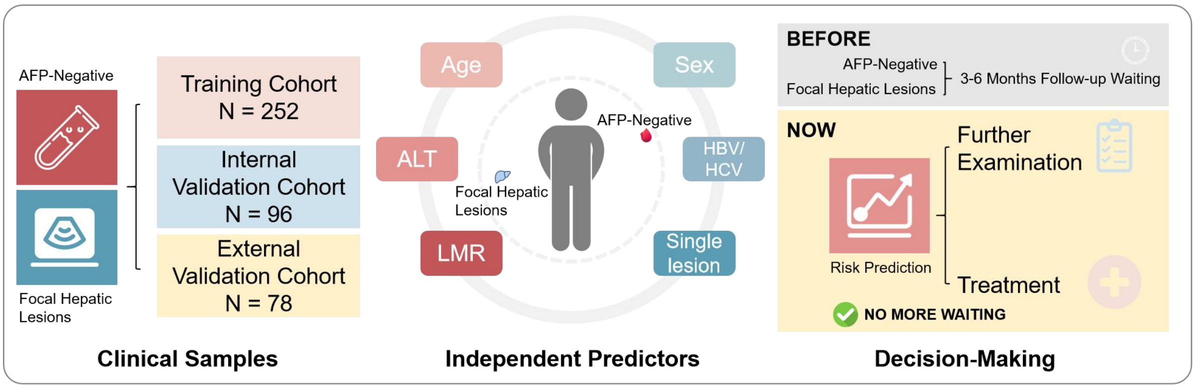

Graph Abstract Patients with liver-occupying lesions who were AFP-negative were divided into training, validation, and external validation sets. Independent risk factors, including age, gender, ALT levels, HBV/HCV infection status, LMR, and single lesion presence, were identified to construct a predictive model. This model aims to reduce the 3-6 month waiting time for follow-up and facilitate rapid clinical decision-making.
